# Photocatalytic Magnetic Microgyroscopes with Activity-Tunable Precessional Dynamics

**DOI:** 10.1021/acs.nanolett.4c03386

**Published:** 2024-11-11

**Authors:** Dolachai Boniface, Arthur V. Straube, Pietro Tierno

**Affiliations:** † Departament de Física de la Matèria Condensada, Universitat de Barcelona, Av. Diagonal 647, 08028 Barcelona, Spain; ‡ Zuse Institute Berlin, Takustraße 7, 14195 Berlin, Germany; ¶ Department of Mathematics and Computer Science, Freie Universität Berlin, Arnimallee 6, 14195 Berlin, Germany; § Institut de Nanociència i Nanotecnologia, Universitat de Barcelona, 08028 Barcelona, Spain; ∥ Universitat de Barcelona Institute of Complex Systems (UBICS), Universitat de Barcelona, 08028 Barcelona, Spain

**Keywords:** Active Colloids, Magnetism, Chemophoresis, Osmosis

## Abstract

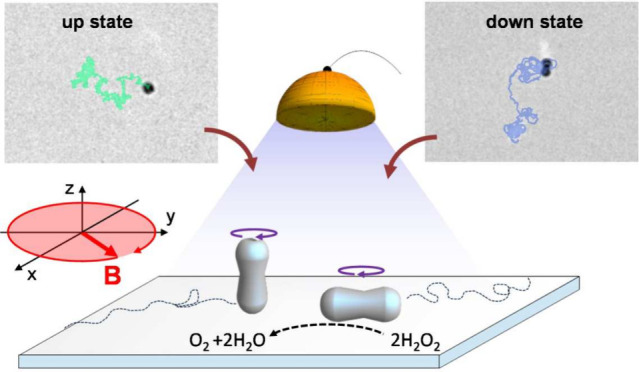

Magnetic nano/microrotors are passive elements spinning around an axis due to an external rotating field while remaining confined to a plane. They have been used to date in different applications related to fluid mixing, drug delivery, or biomedicine. Here we realize an active version of a magnetic microgyroscope which is simultaneously driven by a photoactivated catalytic reaction and a rotating magnetic field. We investigate the uplift dynamics of this colloidal spinner when it precesses around its long axis while self-propelling due to the light induced decomposition of hydrogen peroxide in water. By combining experiments with theory, we show that activity emerging from the cooperative action of phoretic and osmotic forces effectively increases the gravitational torque, which counteracts the magnetic and viscous ones, and carefully measure its contribution. Finally, we demonstrate that by modulating the field amplitude, one can induce hysteresis loops in the uplift dynamics of the spinners.

Spinning tops are mechanical anisotropic objects able of defying gravity while spinning around their vertical axis on a tiny tip.^1^ When actuated by a twisting force, the tops can rise at a vertical position and rotate slowly around an axis until friction and dissipation inevitably terminate their motion. Spinning tops are also part of mechanical gyroscopes, important for navigational systems^2,3^ and used as precise inertial sensors.^1^ Thus, the macroscopic precession of anisotropic systems has been a subject of extensive research to date.

Recent years have witnessed an increasing interest in investigating the spinning motion of driven micro/nanoscale particles, due to their direct application in disparate technological fields including microfluidics,^4−6^ microrheology,^7,8^ sensors^9,10^ and biotechnology.^11^ Experimental realizations of precessing magnetic stirrers at such scale include ferromagnetic nanorods^12^ or magnetic Janus colloids,^13,14^ and they have been recently used to investigate the entropy-driven thermal reorientation of a single element^14^ or the collective self-assembly process.^15^ However, all these cases involve passive particles in the absence of self-propulsion, which prevents the rotating element from moving and stirring across the plane.

In this context, ferromagnetic nanorods have been used in the past as microrheological tools^16−21^ or to create localized microvortices able to trap^22−24^ and stir^25,26^ nonmagnetic tracer particles. Introducing activity via self-propulsion may lead to rich dynamic states with additional functionality. For example, an active magnetic rotor will be able to translate and does not need to stop its rotational motion within the dispersing medium. In fact, passive micro/nanostirrers lack translational motion, which could only be induced by changing the plane of rotation of the applied field, i.e., when it rotates perpendicular to the sample plane^23^ such that these particles stop stirring and behave as surface rotors.^27−29^

**Figure 1:**
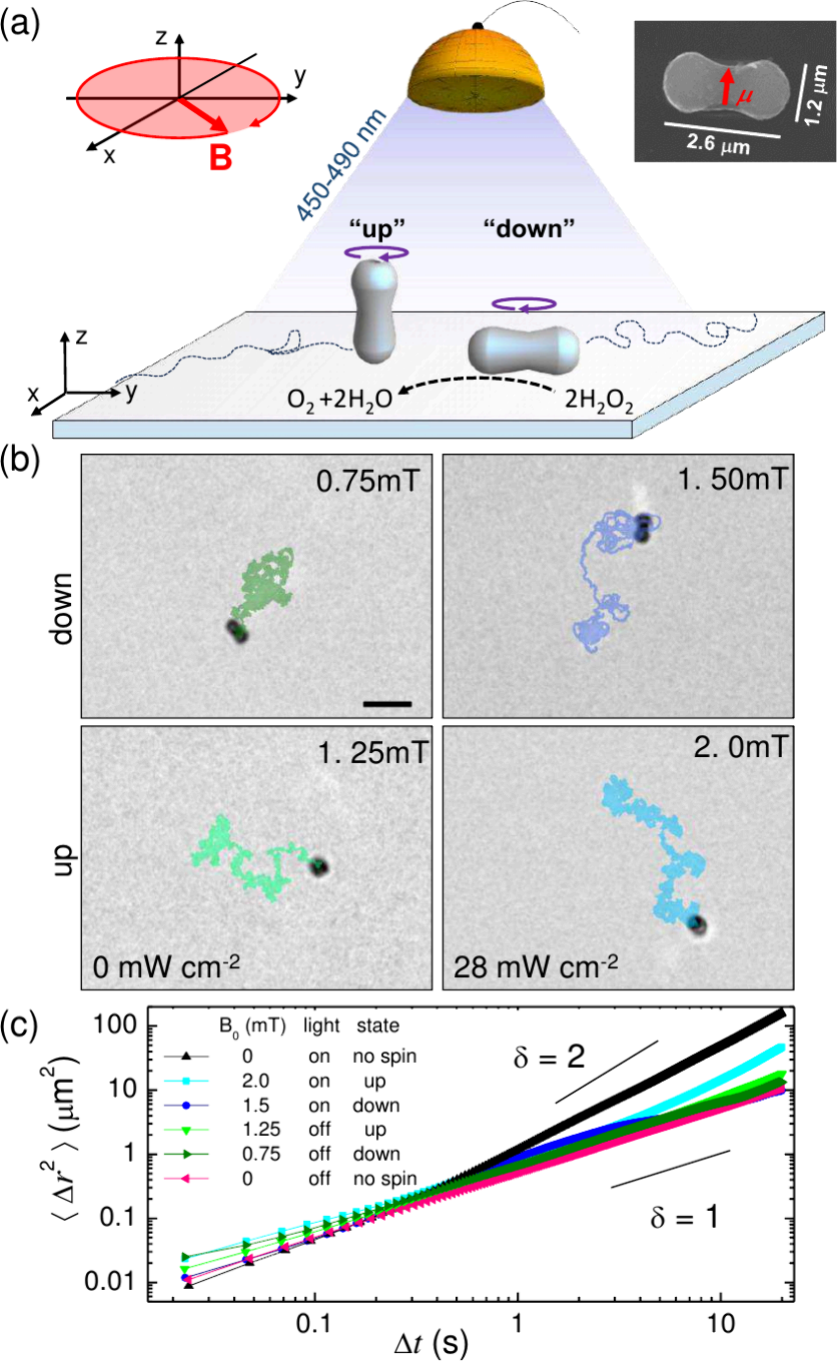
(a) Schematic showing the two dynamic states, “up” and “down”, of a hematite particle under the combined action of a rotating magnetic field **B** and the light induced decomposition of hydrogen peroxide (H_2_O_2_) in water. The small inset at the top right shows the scanning electron microscope image of one hematite particle with the permanent moment **μ** superimposed. (b) Optical microscope images of the hematite particle in the down (top row) and up (bottom row) states in the absence (first column) and in the presence (second column) of blue light with the trajectories overlaid. The number at the top of each image is the field amplitude of the rotating field, scale bar in the first image is 5 μm. The corresponding video (Video S1) can be found in the Supporting Information. (c) Translational mean squared displacement ⟨Δ*r*^2^⟩ versus lag-time Δ*t* for spinning rotors in different dynamic states.

Our active microgyroscopes are made of ferromagnetic hematite particles with a peanut shape, as shown in the small inset in Figure 1a. This particular shape results from the sol–gel process used to synthesize them.^30^ The fabricated particles are characterized by two connected lobes with a long (short) axis equal to *a* = 2.6 μm (*b* = 1.2 μm). As shown in the scanning electron microscope images in Figure 6 of the Supporting Information, the synthesized particles display a rather low polydispersity on the order of ∼5%. The colloidal particles are made of hematite, which is a metal-oxide-based semiconductor characterized by a narrow bandgap energy of 2.1 eV. Due to this bandgap, light can be absorbed within the visible region up to 570 nm. Thus, in the presence of blue light, the hematite harvests enough energy to power the H_2_O oxidation. In particular, as previously reported,^31,32^ when exposed to blue light, hematite colloids can act as catalysts triggering the chemical decomposition of diluted H_2_O_2_ in water and display self-propulsion. In contrast, under only white light from an optical microscope, the particles do not show any directed motion apart from the thermal fluctuations, even in the presence of H_2_O_2_. The light-induced chemical reaction is given by 2H_2_O_2(l)_ → O_2(g)_ + 2H_2_O_(l)_. This reaction creates a chemical concentration gradient around the particle surface, inducing diffusiophoresis. Due to this phenomenon, the hematite particle can attract passive colloids nearby^33^ and display self-propulsion.^34^

We disperse the particles in an aqueous basic (pH = 9) solution containing 3.6% by volume of H_2_O_2_, which is enclosed within a rectangular glass microtube (inner dimension 2 × 0.1 mm). After a few minutes, the hematite particles sediment nearby to the bottom plate due to density mismatch and display diffusive dynamics. We induce self-propulsion by applying blue light at wavelength λ = 450–490 nm with a tunable intensity *I* ∈ [0, 125] mW cm^–2^. Moreover, before the experiments, we employ an etching treatment with hydrochloric acid to enhance the particle activity.^34^ Additionally, we applied an external rotating magnetic field to induce spinning motion. This is possible since the hematite particles are slightly ferromagnetic and display a small permanent dipole moment μ = 2 × 10^–16^ A m^2^ oriented along their short axis,^35^Figure 1a. The presence of a permanent magnetic moment perpendicular to the particle long axis is due to the magnetic structure of hematite, which crystallizes in the corundum form.^36^ In such a phase, the iron cations are aligned antiferromagnetically along the particle long axis (*c*-axis). However, above the Morin temperature (*T* ∼ 263 K) the hematite transit to a ferromagnetic state where the magnetic spins are aligned along the basal plane, which is perpendicular to the *c*-axis. More technical details on the experimental protocol, the particle magnetic properties, and the experimental setup can be found in Section 1 of the Supporting Information.

We apply a rotating magnetic field circularly polarized in a plane (***x̂***, ***ŷ***) parallel to the glass substrate:
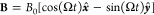
1with *B*_0_ the field amplitude and Ω = 2*πf*, where *f* is the driving frequency. The hematite particles perform a spinning motion around their short axis with a frequency *f*_s_, and at a fixed field amplitude *B*_0_ = 1 mT, they rotate parallel to the bounding plane for driving frequencies *f* < 4.5 Hz, which we refer to as the “down” state. In contrast, for *f* ∈ [4.5, 10] Hz, the particles preferentially stand up, performing a spinning motion around their long axis, thus perpendicular to the glass substrate, the “up” state; see Figure 1a. Note that the frequency range of the up state increases with the field amplitude, with *f* ∈ [4.5, 20] Hz for *B*_0_ = 2 mT and *f* ∈ [4.5, 31] Hz for *B*_0_ = 3 mT. These two dynamic states can be controlled by adjusting *B*_0_ and *f*, in addition to which we include the light activation, making these spinning rotors self-propelling. Figure 1b displays typical trajectories of the hematite rotors in four possible situations: when they are in the down (top row) and up (bottom row) states and in the presence of light (right column) or in the absence of it (left column).

In the absence of light, *I* = 0, the rotating hematite particles perform standard diffusive dynamics standing up or lying down, as shown by the measured translational mean squared displacement (MSD) in Figure 1c. Here we calculate the MSD as ⟨Δ**r**^2^(Δ*t*)⟩ ≡ ⟨(**r**(*t* + Δ*t*) – **r**(*t*) )^2^⟩ ∼ Δ*t*^δ^, with **r**(*t*) the position of the particle center at time *t*, Δ*t* the lag time, and ⟨...⟩ a time average. The MSD can be used to distinguish between the normal diffusive (δ = 1) dynamics from the sub[super] diffusive (δ < 1 [δ > 1]) and ballistic (δ = 2) ones. When the particles are activated by light (*I* = 28 mW cm^–2^), we find that in the absence of spinning (*B*_0_ = 0), the active particle exhibits a ballistic trajectory after a few seconds with δ ∼ 2. In contrast, the effect of spinning is to localize the trajectories, reducing the corresponding exponent in the MSD. Specifically, we observe diffusive behavior for spinning down and superdiffusive, almost ballistic behavior for spinning up. This effect can be understood by observing that a rotating object in a viscous fluid generates an hydrodynamic flow field,^37^ which in first approximation is purely azimuthal and decays as ∼1/*r*^2^.^38^ Such a flow may transport away the product of the catalytic reaction, reducing thus the concentration gradient around the particle and thereby the corresponding self-propulsion.^39−41^

To gain insights into the different dynamic states, we have modified the model proposed in ref (12) to our situation, by incorporating the additional effect of the catalytic reaction and the different geometry (shape). We approximate the particle as an ellipsoid with the major axis *a* and minor axis *b*, mass *m*, and permanent moment **μ** oriented along the minor axis and suspended in liquid medium of dynamic viscosity η. Further, as shown in Figure 2a, we have used two coordinate systems, the laboratory one (***x̂***, ***ŷ***, ***ẑ***) and a second one that is fixed to the ellipsoid (***ê***_1_, ***ê***_2_, ***ê***_3_) with ***ê***_1_ and ***ê***_3_ aligned with **μ** and the long axis of the ellipsoid, respectively. Both reference frames are connected through the Euler angles (ϑ, ϕ, ψ). In the absence of chemical activity, the overdamped motion of the ellipsoid is governed by the balance of different torques: magnetic, **τ**_*m*_ = **μ** × **B**, gravitational, , and viscous, , torques:
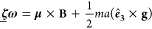
2where **g** is the gravitational acceleration, **ω** is the angular velocity, and **ζ** = diag(ζ_1_, ζ_2_, ζ_3_), where ζ_1_ = ζ_2_ ≠ ζ_3_ is the rotational friction tensor in the main axes. Our main hypothesis is that the chemical activity generates an attractive phoretic/osmotic force **F**_p/o_ between the hematite and the substrate. This force gives rise to a corresponding torque,  which prevents the hematite from uplifting, inducing an effective increase of the gravitational torque **τ**_*↓*_ = **τ**_p/o_ + **τ**_g_. Also, since the hematite is pressed against the substrate, there is a rise in the drag torque which now becomes **τ**_d_. To take into account the effect of these osmotic/phoretic contributions avoiding the complex details of the corresponding chemical reactions, we introduce the dimensionless prefactor α ⩾ 1, β ⩾ 1,
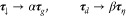
such that α = 1, β = 1, for the system without activity.

**Figure 2:**
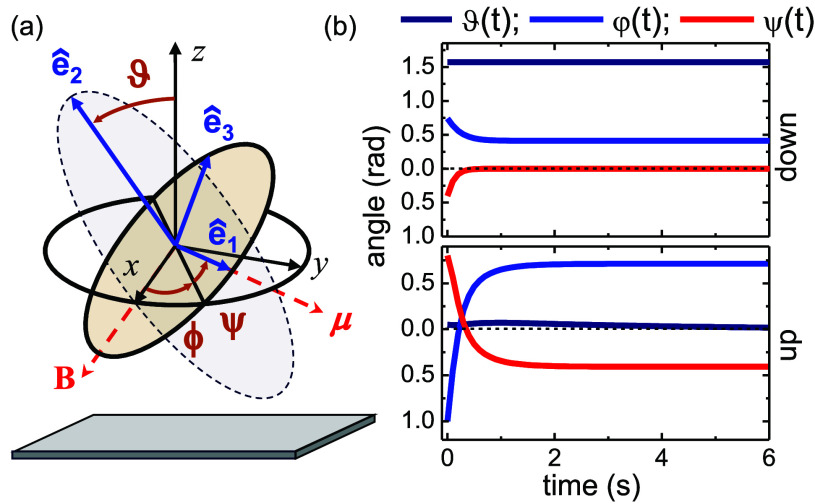
(a) Schematic showing an elliptical particle (dashed line) with the laboratory reference frame (***x̂***, ***ŷ***, ***ẑ***), the coordinate system fixed on the particle (***ê***_1_, ***ê***_2_, ***ê***_3_), the three Euler angles (ϑ, ϕ, ψ), and the orientation of the applied magnetic field **B** and magnetic moment **μ**. (b) Time evolution of the Euler angles from eqs 3a and 3b for *B̂* = 5.0 and at two different driving frequencies, Ω̂ = 2.0 (down state, top panel, note ϑ = π/2) and Ω̂ = 3.0 (up state, bottom panel, note ϑ = 0).

Introducing dimensionless time, , frequency, , and field, , we arrive at the governing equations of motion:
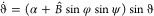
3a
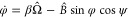
3b
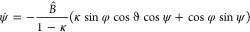
3cHere, κ = (ζ_1_ – ζ_3_)/ζ_1_ specifies the particle’s asymmetry and we have introduced the angle φ = ϕ – Ω*t*, implying the transition to the reference frame rotating together with the external field. The cases with ϑ = π/2 and ϑ = 0 describe the down and up states, respectively. For α = 1, β = 1, eq 3 corresponds to the passive system,^12^ when the blue light and hence the activity are switched off. Figure 2b shows that eq 3 correctly predicts the two dynamic states experimentally observed by varying the field parameters. For a fixed value of the rescaled field, *B̂* = 5.0 the stable state is the rod lying down (ϑ = π/2) for a low frequency, Ω̂ = 2.0 (top panel), or standing up (ϑ = 0) for a large one, Ω̂ = 3.0 (bottom panel).

We first characterize with experiments how the particle dynamics are influenced independently by the magnetic field (Figure 3a) and by the activity (Figure 3b,c). At low frequencies, we observe the down state. Such a state, where ϑ = π/2, ψ = 0 is described by the single simplified equation , cf. eq 3b. The latter equation predicts two dynamic regimes separated by critical frequency *f*_c_. Formulated in the dimensional units relative to the laboratory reference frame, for *f* < *f*_c_ the particle rotates synchronously with **B**, and its average spinning frequency ⟨*f*_s_⟩ = *f*, as shown by the black squares in Figure 3a. In contrast, for *f* > *f*_c_ the particle still spins but does it in an asynchronous regime, where the average spinning drops down as ; see the disks in Figure 3a. These two regimes are connected by the critical frequency *f*_c_ which, as shown in the small inset in Figure 3a, scales linearly with the applied field as *f*_c_ = *μB*_0_/(2*πζ*_1_). The data in such an inset have been extrapolated from the nonlinear regressions, since for a range of intermediate frequencies the hematite particles were observed to enter in the up state rather than remain confined to the close plane. In the up state, the spinning particle appears under the microscope as a circular disk, as shown in the bottom images of Figure 1b. This effect makes it difficult to precisely extract the particle orientation and thus to determine *f*_s_ from the particle tracking. The corresponding frequency domains are highlighted by the arrows within the shaded regions in the graph in Figure 3a.

**Figure 3:**
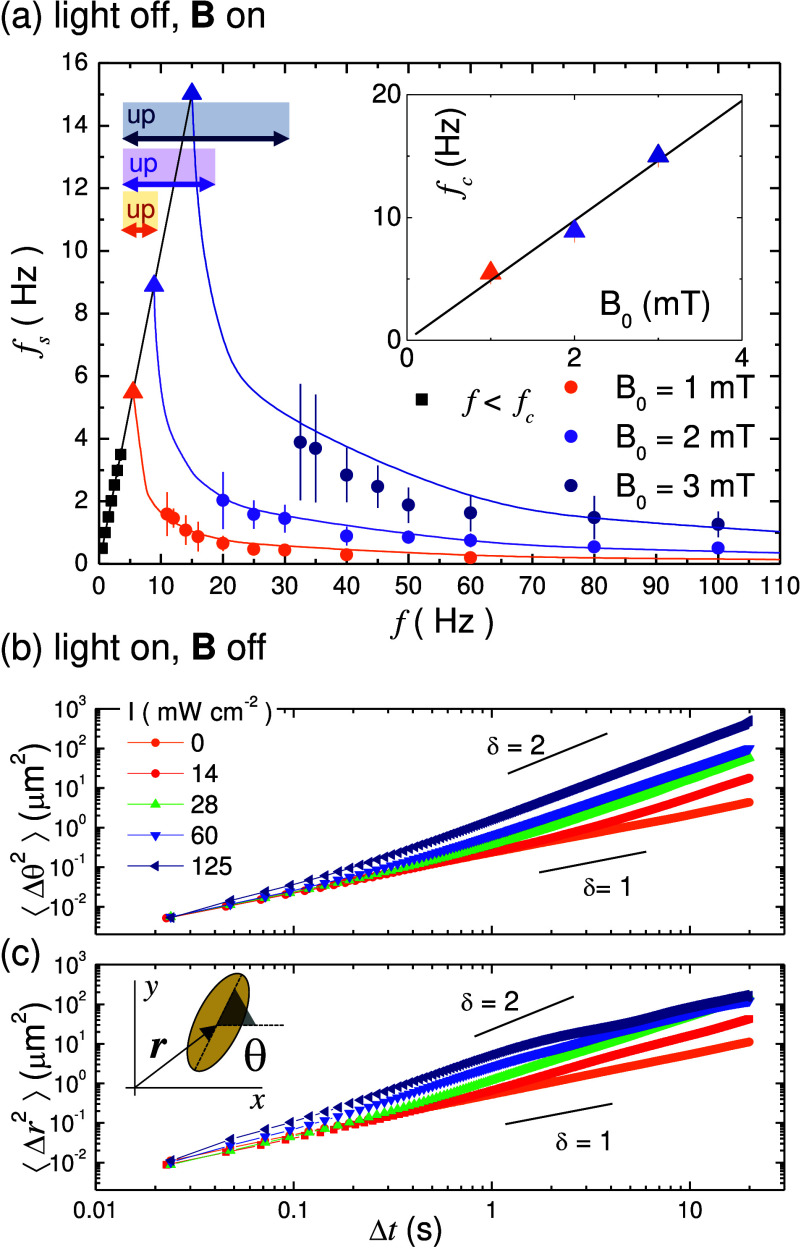
(a) Spinning frequency *f*_s_ of a single hematite particle versus field frequency *f* for three field amplitudes *B*_0_. Scattered symbols are experimental data; continuous lines are nonlinear regressions of the synchronous (*f* < *f*_c_) and asynchronous (*f* > *f*_c_) regimes. The triangles indicate the values of *f*_c_ extracted from these curves and are shown in the top inset. The arrows within the shaded regions in the graph indicate that the particle is in the up state. (b,c) Angular ⟨Δθ^2^⟩ (b) and translation ⟨Δ*r*^2^⟩ (c) mean squared displacements of a hematite particle in the absence of magnetic field and under different light amplitudes *I*.

Our magnetic rotors can also be activated by blue light. In Figure 3b, we show the effect of the light induced decomposition of H_2_O_2_ by measuring both the angular MSD, ⟨Δθ^2^⟩ (top), and the translational one, ⟨Δ**r**^2^⟩ (bottom). In the absence of light (*I* = 0), both MSDs show diffusive dynamics with a long-time rotational diffusion coefficient *D*_θ_ = 0.140 ± 0.001 rad^2^ s^–1^ and a translational one, *D*_**r**_ = 0.152 ± 0.001 μm^2^ s^–1^. However, when the light is on, the hematite particle becomes activated and displays a superdiffusive dynamics. This effect strongly emerges in the angular dynamics, where we initially observe a diffusive behavior followed by a superdiffusive/ballistic behavior at large Δ*t*. The threshold between both dynamic states decreases with the light amplitude *I*, and for the maximum power (*I* = 125 mW cm^–2^) it reduces to Δ*t* ∼ 0.1 s. The activity also affects the translational MSD, as shown in Figure 3c. In particular, we observe that for small light intensities (*I* = 14, 28, 60 mW cm^–2^) the behavior is similar to the angular MSD with an initial diffusive regime (δ = 1) followed by a superdiffusive one (δ > 1). In contrast, at maximum light power and thus activity, *I* = 125 mW cm^–2^, the behavior becomes the opposite, and the trajectory localizes more. The particle displays first an initial diffusive behavior which is immediately followed, after Δ*t* = 0.2 s, by a superdiffusive one and then switches back to a diffusive regime at long time, Δ*t* > 5 s. In this case, we observe an enhanced diffusion with a coefficient that is more than 10 times higher than the passive case, *D*_**r**_ ∼ 2 μm^2^ s^–1^. This feature results from the etching process of the particles (see Section 2 of the Supporting Information), which roughens the particle surface, enhancing the generated chemophoretic flow and the corresponding self-propulsion behavior. Also, we have independently checked that the illumination intensities used in our work did not induce any change in the temperature of the system that could alter the particle dynamics. Indeed, in a separate set of experiments, we have tracked the diffusive motion of 2 μm size silica spheres, at different applied powers, and obtained similar diffusion coefficients at all light intensities.

We now consider the combined effect of the activity and spinning on the particle dynamics. The catalytic reaction induces self-propulsion due to a local gradient, triggering osmotic and phoretic phenomena. Also, this reaction creates an effective attractive force between the hematite particle and the substrate. This force tends to elevate the threshold field  required for the particle to transit toward the up state. We confirm this effect in Figure 4a, where we have measured  by varying the light intensity, and thus the activity, in the (*B*_0_, *f*) plane. Thus, the blue light shifts the limit  toward higher magnetic amplitudes. From the model given by eq 3a, it follows that the up and down states start to coexist at a critical field value,
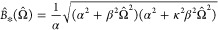
4

**Figure 4:**
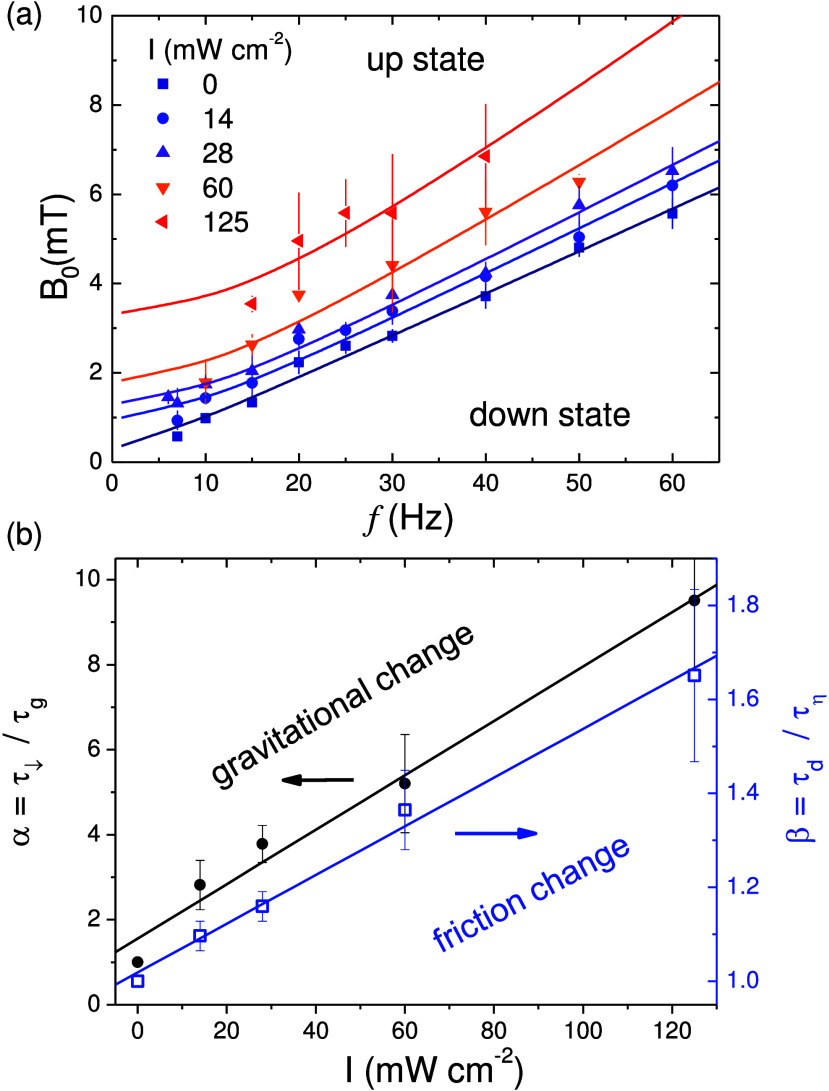
(a) Border between the up and down states in the (*f*, *B*_0_) plane and for different light intensities *I*. Scattered symbols are experimental data while continuous lines are nonlinear regressions from eq 4. (b) Evolution of the parameter α (black), indicating the raise of the gravitational-like torque (**τ**_↓_) due to the light intensity *I*, and β (blue), which corresponds to the variation of the drag torque (**τ**_d_).

The complete derivation of this equation is given in Section 3 of the Supporting Information. Equation 4 shows that, at low κ, changing α will shift the critical magnetic field amplitude, while changing β will alter the slope of the curve for sufficiently small Ω. In the latter case, we specifically verify this effect by increasing the viscosity of the solution through the addition of glycerol. In this specific scenario, we vary β = η/η_w_, where η is the viscosity of the solution and η_w_ that of water, as shown in Section 4 of the Supporting Information.

We use eq 4 to fit the experimental data describing the uplift transition induced by the rotational motion, Figure 4a. First we consider the transition in the absence of activity, *I* = 0. Thus, we fix α = 1, β = 1 and determine the remaining experimental parameter, here κ = 0.0076, and the rescaling prefactors. Next, we fix κ and change both α and β by performing the simultaneous regression of all the experimental data obtained at different *I*. The multiple regressions confirm the good agreement between the model and the experimental data. More importantly, this agreement highlights that the effect of the activity effectively increases the vertical force pushing the particle towards the substrate. Even in presence of this attraction, the uplift force arising from the magnetic torque allows us to precisely measure this contribution. Indeed, from the variation of  it follows that α, which represents the intensity of the attractive force relative to the gravity, increases linearly with the light intensity. This phoretic/osmotic force**F**_p/o_ increases the gravitational one from up to 10 times for the maximum light intensity, *I* = 125 mW cm^–2^ and, correspondingly, the light induced torque **τ**_***↓***_ ∝ **τ**_*g*_, Figure 4b. Concerning the quantity β, which can be considered as a measure of the variation of the drag torque, it also increases with the light intensity but less drastically, as shown by the blue line in Figure 4b. This effect is due to the fact that the attractive phoretic force pushes the hematite particle close to the substrate, thus raising the hydrodynamic drag coefficients.

We finally comment on the origin of the chemically induced force **F**_p/o_ = **F**_p_ + **F**_o_, which results from both diffusiophoresis (**F**_p_) and diffusioosmosis (**F**_o_) contributions. Diffusioosmosis is the spontaneous flow of solutes along a solid surface under a concentration gradient, while diffusiophoresis is the spontaneous motion of particles under a concentration gradient of solutes. Classically, diffusiophoresis is viewed as an effect of diffusioosmotic flow arising from a surface concentration gradient along the particles,^42^ while the osmotic flow tends to push the particle along the opposite direction.^43^ The surface concentration gradient ∇_∥_c, along the hematite particle or substrate, generates a slip velocity *u*_slip_ ∝∇_**∥**_c, leading to viscous forces acting on the hematite^44^ due to the generated hydrodynamic flow fields. Both components tend to push the particle toward the surface. The phoretic effect has been observed in previous works^31,45^ to induce an attraction between passive objects, as the substrate, and photocatalytic active hematite particles. For the osmotic component, the chemical activity of hematite generates a radial concentration gradient along the substrate. The slip velocity over the substrate initiates a centripetal osmotic flow.^44^ Due to the incompressibility of water, a vertical flow compensates for the radial flow, pushing the hematite against the substrate through a viscous interaction. To support our reasoning, in Section 5 of the Supporting Information we offer a minimal model that justifies this vertical force. The model approximates the particle as a sphere with a point source of chemical at the center and predicts a vertical force that may exceed the gravitational one by an order of magnitude, as observed in the experiments.

The uplift dynamics of our active magnetic microgyroscopes can display hysteretic behavior, as shown in Figure 5. This effect is induced by periodically varying the field amplitude via a triangular wave while keeping constant the driving frequency of *f* = 10 Hz. We use a triangular function with a period *T* = 120 s, such that it starts at zero and reaches linearly the maximum at 60 s and then returns linearly to *B*_0_ = 0 mT at the end of the cycle; see the bottom inset of Figure 5. We quantify whether the spinning particle is in the up or down state during the field cycle by measuring the eccentricity *E* of the observed area of the hematite particle from video-microscopy. Thus, the particle is in the up (down) state if *E* ≳ 0.8 (*E* ≲ 0.3). We observe a sharp transition from the down to the up state around a critical amplitude, *B*_c_, and a hysteresis between the rising and falling part of the cycle each associated with a different critical field amplitude. As shown in Figure 5 by the solid bold lines, these experimental curves are fitted using classical models for magnetic hysteresis.^46^ Without light (top panel), the critical amplitude is *B*_c_ ∼ 1.5 mT, with a gap in the hysteresis cycle of ∼ 0.25 mT. With blue light at *I* = 14 mW cm^–2^ (bottom panel), the critical amplitude is shifted toward a higher value *B*_c_ ∼ 2 mT), and the gap is doubled. We also note that the transition for the decreasing states remains almost at the same field amplitude with or without light.

**Figure 5:**
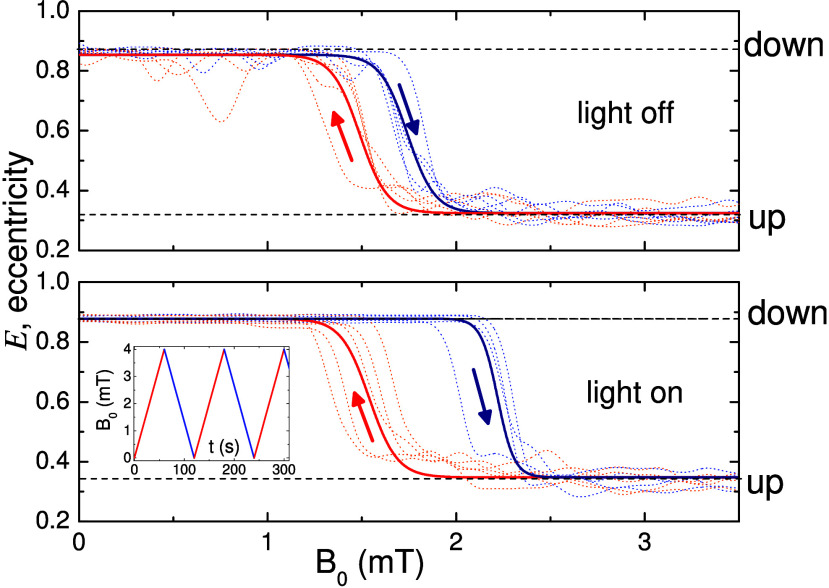
Hysteresis loops of the particle eccentricity *E* versus the applied magnetic field in the absence of light (top) and with blue light *I* = 14 mW cm^–2^ (bottom). Dotted lines are experimental data, while bold solid lines result from a nonlinear regression using the following equation: , with *a*_1,2_ and *b*_1,2_ fitting parameters. The bottom inset shows the triangular modulation of the field amplitude.

In conclusion, we have experimentally realized an active magnetic microgyroscope, where spinning and activity are simultaneously and independently controlled by two different actuating fields. We investigate how spinning affects the microgyroscope transport and find that the rotational motion strongly suppresses self-propulsion compared with the zero magnetic field case. We then investigate the uplift dynamics when these microgyroscopes are subjected to an in-plane rotating field and stand up to reduce viscous dissipation. In particular, by balancing all torques acting on the rotating particles, we find that the activity induced by a catalytic chemical reaction can be considered as an additional gravitational-like torque, which increases the amplitude threshold field to transit the particle in the up state. We use a theoretical model to capture the basic physics of the process by balancing magnetism, gravity, viscosity, and activity. These spinning self-propelling agents can be used in microfluidic channels as active component, adding further feasibility to previous experimental realizations based on passive (i.e., nonactive) colloidal rotors.^47,48^

## Supporting Information Available

Experimental methods, setup, magnetic properties, derivation of eq 4, viscosity measurements, vertical force discussion, theoretical model, and figures supporting this information (PDF)
Dynamics of the spinning hematite colloids (MP4)
