# Supplementary material for: Photocatalytic Magnetic Microgyroscopes with Activity-Tunable Precessional Dynamics
Source: Nano Lett. 2024 Nov 11;24(47):14950–6. doi: 10.1021/acs.nanolett.4c03386 (PMC11613690; doi:10.1021/acs.nanolett.4c03386)
Supplement: Supplementary file 1 — nl4c03386_si_001.pdf [file nl4c03386_si_001.pdf]

Supporting Information for: Photocatalytic magnetic microgyroscopes with activity-tunable precessional dynamics

Dolachai Boniface,[†] Arthur V. Straube,[‡,¶] and Pietro Tierno[*,†,§,‖]

†Departament de Física de la Matèria Condensada, Universitat de Barcelona, Av. Diagonal 647, 08028 Barcelona, Spain

‡Zuse Institute Berlin, Takustraße 7, 14195 Berlin, Germany

¶Freie Universität Berlin, Department of Mathematics and Computer Science, Arnimallee 6, 14195 Berlin, Germany

§Institut de Nanociència i Nanotecnologia, Universitat de Barcelona, 08028, Barcelona, Spain

‖Universitat de Barcelona Institute of Complex Systems (UBICS), Universitat de Barcelona, 08028, Barcelona, Spain

E-mail: ptierno@ub.edu

Section S1: Experimental system

Particle synthesis, etching and sample preparation

We synthesize the anisotropic hematite particles following the "gel-sol" technique.[1] In particular, we mix during stirring a solution made of $54.0\,\mathrm{g}$ of iron chloride hexahydrate (Sigma-Aldrich 31232-M) in $100\,\mathrm{mL}$ of deionized water with another solution composed of $19.48\,\mathrm{g}$ of sodium hydroxide (Sigma-Aldrich S5881) in $90\,\mathrm{mL}$ of water. After ~ 5 minutes, we add gradually a solution made of $0.86\,\mathrm{g}$ of potassium sulfate (Sigma-Aldrich P0772) in $10\,\mathrm{mL}$ of water. Compared

to a previous work that produced an ellipsoidal shape,[2] the peanut shape is obtained with a higher concentration in potassium sulfate. After stirring for 5 more minutes, the mixture is hermetically sealed in a 1L bottle and left to age at $100°$C for 8 days.

Afterwards, the reaction is stopped by filling the bottle with highly deionized water and leaving it to cool down in a fridge. The hematites are concentrated and washed through multiple cycles involving centrifugation and re-dispersion in deionized water. The final result is a dispersion of hematite colloids with a peanut shape as shown in the two scanning electron microscope images in Fig. 6(a).

On the day of the experiment, $500\,\mu$L of the hematite dispersion is placed in a plastic tube (Eppendorf), and mixed there with $500\,\mu$L of hydrochloric acid (Sigma Aldrich, 37% w/w) at $12.2\,$M. The resulting solution is agitated in a stirrer for $\sim 10\,$min, before being diluted in $15\,$mL of deionized water to reduce the pH and stop the etching process. The hematite particles are then recovered through three cycles of centrifugation and re-dilution processes. As commented in a previous work,[3] the etching treatment drastically increases the diffusio- osmotic/phoretic phenomena. Indeed, in absence of etching, the hematite particles were unable to exhibit self-propulsion.[2]

The experimental sample consists of hematite particles dispersed in a hydrogen peroxide (Fisher BP2633) aqueous solution at a concentration of 3.6 % w/v. To prevent the interactions between the particles, we use a low concentration of hematite such that the average surface area is ~ 400 colloids/mm^2. A small amount of 5 μm silica spheres are added to the dispersion to assist in finding the sedimented layer of colloids. The solution is basified (pH $\sim$ 9.2) by adding Tetramethylammonium hydroxide (Sigma-Aldrich 328251), to prevent the colloids from sticking to the substrate. We leave the solution for $5\,$min in an ultrasound bath to break the hematite chains, which tend to form spontaneously with time due to the particle ferromagnetic nature.

Experimental setup

To visualize the hematite particles, we use an upright optical microscope (Eclipse Ni; Nikon) equipped with a charge coupled device camera (Basler Scout scA640-74f) working at 12 frames

per second, and an epifluorescent tower. For illumination, a commercial mercury fiber illuminator system (C-HGFI Intensilight; Nikon) provides the white light, which passes through a bandpass filter (Nikon B-2A filter) to obtain the blue light ($450 - 490\,\mathrm{nm}$). The light emerging from the microscope objective (Nikon MRH01902) has an intensity that can be tuned from 4 up to $125\,\mathrm{mW\,cm^{-2}}$ as measured by a power meter (Thorlabs).

The magnetic setup is composed of two pairs of perpendicular coils, each aligned along one of the two axis parallel to the glass plate. Two power amplifiers (KEPCO BOP) provide the sinusoidal currents, both controlled by an arbitrary waveform generator (TGA1244, TTi). We ensure that the two currents have the same frequency and a constant $90°$ phase delay between them. A low resistor (0.6 ohms) is placed in series with the coils. After calibration using a teslameter (FM 205, Projekt Elektronik GmbH), the measurement of the voltage across the resistor indirectly provides the magnetic field amplitude B_0.

Section S2: Magnetic properties of the hematite particles.

Here, we provide more details on the magnetic properties of the hematite particles. To measure the strength of their magnetic moment $\boldsymbol{\mu}$, we have performed in the past[4] different experiments by measuring the reorientation dynamics of a single hematite particle under a constant magnetic field, as shown in the schematic in Fig. 6(b). Due to the applied field $\boldsymbol{B}$, the hematite particle is subjected to a magnetic torque, $\boldsymbol{\tau}_m = \boldsymbol{\mu} \times \boldsymbol{B}$, which can be balanced by the viscous torque arising from its rotation in the fluid, $\boldsymbol{\tau}_v = -\zeta_r \dot{\boldsymbol{\theta}}$, with ζ_r the rotational friction coefficient which can be considered, in first approximation, similar to that of an ellipsoid. In the overdamped limit, the torque balance equation becomes: $\boldsymbol{\tau}_v + \boldsymbol{\tau}_m = \boldsymbol{0}$. This equation can be exactly solved, giving:

$$\theta = 2\tan^{-1}\left[\tanh\left(\frac{t}{\tau_r}\right)\right] \tag{1}$$

with $\tau_r = 2\zeta_r/(\mu B)$ the relaxation time. Using the rotational friction coefficient for a prolate ellipsoid,[4] one can fit the experimental data with Eq. (1) to obtain a permanent moment $\mu =$

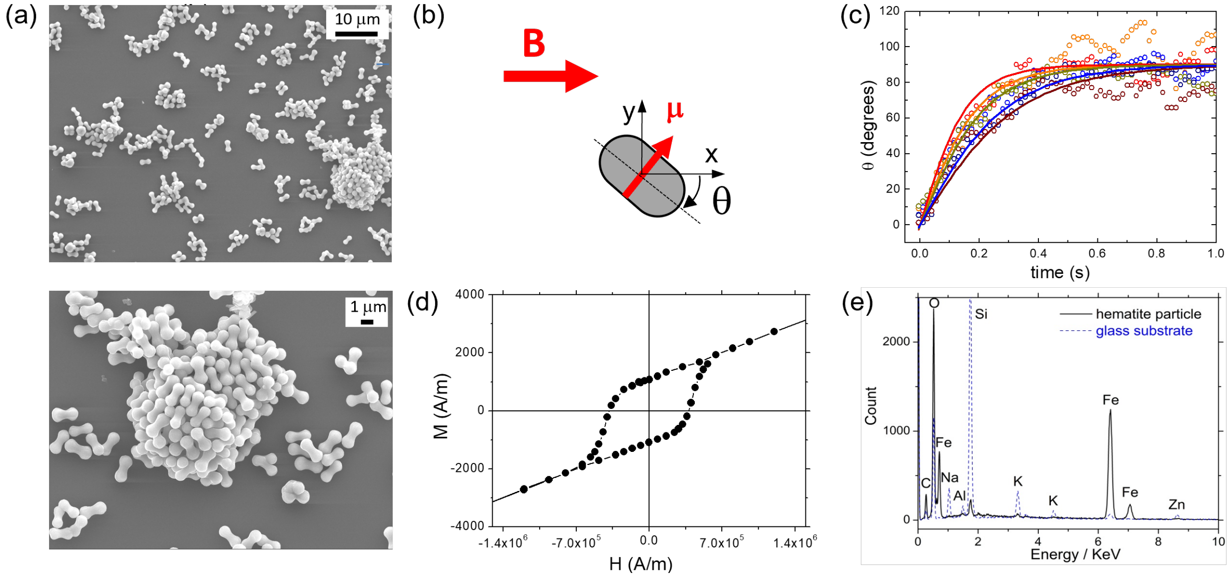

Figure 6: (a) Two scanning electron microscope images with different resolutions of the synthesized hematite particles showing the system monodispersity. (b) Schematic showing the reorientation of a magnetic hematite particle with moment $\boldsymbol{\mu}$ under a constant magnetic field $\boldsymbol{B}$. (c) Corresponding measurement of the relaxation angle θ versus time t. Scattered circles are experimental data while continuous lines are non-linear regression using Eq.(1). (d) Magnetization curve of a dry sample of hematite particles using a Superconducting Quantum Interference Device. (e) Energy-dispersive X-ray spectroscopy of hematite particles (continuous black line) with the sample holder (dashed blue lines) made of a borosilicate glass.

2×10^{-16} A m^2 as shown in Fig. 6(c).

The ferromagnetic nature of the hematite particles can be independently confirmed by performing magnetization measurements, as shown in Fig. 6(d) and the presence of magnetic elements in the synthesized particles is also evident from the Energy-dispersive X-ray spectra, shown in Fig. 6(e). In the latter case, the presence of peaks at 0.71, 6.41 and 7.05 KeV indicate the location of Fe, the peaks at 0.52 KeV of Oxygen and that at 1.74 KeV of Si, which come from the sample holder.[5]

Section S3: Derivation of equation (4)

The governing equations given by Eqs. (3) in the main text present the generalization of the model for a passive rod,[6] which refers to the special case of $\alpha = 1$, $\beta = 1$. It was shown that, at a

given field amplitude B_0 (or $\hat{H}$ in terms of Ref. [6]) and for low frequencies, the down state is stable while the up state is unstable. As one increases the frequency, the down state remains stable and the upper state turns stable as well. This happens at a critical frequency $\hat{\Omega}$ determined by the critical curve $\hat{H} = \hat{H}_3(\Omega)$, and the down and up states start to coexist. In the generalized case of $\alpha > 1$, $\beta > 1$, the corresponding critical dependence is given by an analogous generalized condition, $\hat{B} = \hat{B}_*(\hat{\Omega})$. Here, $\hat{B}_*(\hat{\Omega})$ depends also on α and β, as given by Eq. (4) of the main text. We now derive this generalized expression.

Equation (4) of the main text follows as the condition at which the upper stationary state ($\vartheta = 0, \varphi_0, \psi_0$) with $\varphi_0, \psi_0 = \text{const}(t)$ loses its stability. The upper state, $\vartheta = 0$, satisfies Eq. (3a) for any φ_0, ψ_0 and its stability is determined by the sign of the expression in the brackets in Eq. (3a) such that at the border of stability it turns to be zero:

$$\alpha + \hat{B}_* \sin\varphi_0 \sin\psi_0 = 0\,. \tag{2}$$

The values φ_0 and ψ_0 obey stationary Eqs. (3b) and (3c) taken at $\vartheta = 0$, which can be written as

$$\hat{B}_* \sin\varphi_0 \cos\psi_0 = \beta\hat{\Omega}\,,$$

$$\hat{B}_* \cos\varphi_0 \sin\psi_0 = -\kappa\beta\hat{\Omega}\,.$$

Building the sum and the difference of the above equations, one immediately finds that

$$\varphi_0 \pm \psi_0 = \arcsin\left[\frac{(1 \mp \kappa)\beta\hat{\Omega}}{\hat{B}_*}\right]\,. \tag{3}$$

Representing $\sin\varphi_0 \sin\psi_0$ as $[\cos(\varphi_0 - \psi_0) - \cos(\varphi_0 + \psi_0)]/2$ in Eq. (1) and accounting for Eq. (2), we find

$$4\alpha^2 = 4(\hat{B}_* \sin\varphi_0 \sin\psi_0)^2 = \sqrt{\hat{B}_*^2 - (1+\kappa)^2\beta^2\hat{\Omega}^2} - \sqrt{\hat{B}_*^2 - (1-\kappa)^2\beta^2\hat{\Omega}^2}\,.$$

Squaring up of this intermediate result and gathering the terms at different powers of $\hat{B}_*$, we arrive at the expression $\alpha^2 \hat{B}_*^2 = (\alpha^2 + \beta^2 \hat{\Omega}^2)(\alpha^2 + \kappa^2 \beta^2 \hat{\Omega}^2)$ and hence

$$\hat{B}_* = \frac{1}{\alpha} \sqrt{(\alpha^2 + \beta^2 \hat{\Omega}^2)(\alpha^2 + \kappa^2 \beta^2 \hat{\Omega}^2)} \ . \tag{4}$$

Equation (4) here coincides with Eq. (4) in the main text used for fitting, which is a generalization of the corresponding expression for $\alpha > 1$, $\beta > 1$; at $\alpha = 1$, $\beta = 1$ it reduces to the expression for the passive rod (cf. expression for $\hat{H}_3(\hat{\Omega})$ in Ref. [6]).

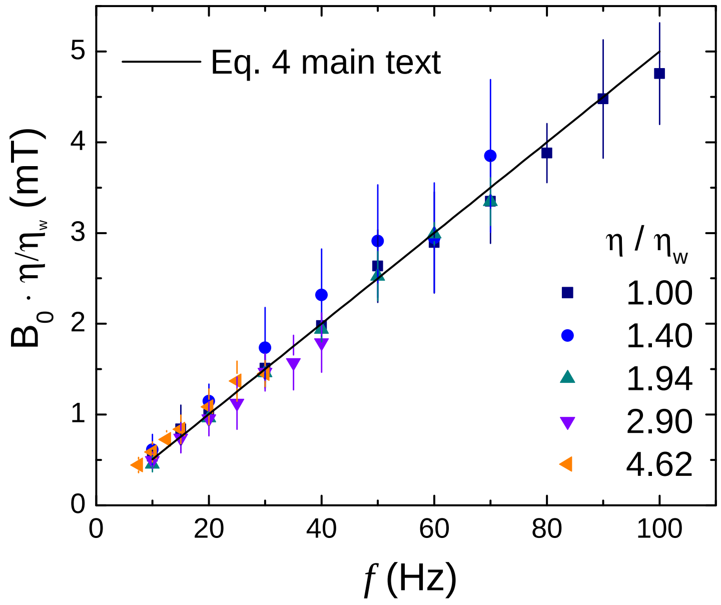

Figure 7: Threshold field B_0 for the transition to stand up multiplied by the rescaled viscosity η/η_w, being $\eta_w = 10^{-3}\mathrm{Pa \cdot s}$ the viscosity of water versus driving frequency f and for different ratios η/η_w. Experimental data are scattered symbols, the continuous black line is a non-linear regression using Eq.(4) of the main text.

Section S4: Effect of the viscosity

Here we test if Eq. (4) predicts the effects induced by varying the viscosity of the solution η, which corresponds to adjusting β, while keeping $\alpha = 1$. To modify the viscosity, we perform several experiments by adding a controlled amount of glycerol to water (viscosity $\eta_w = 10^{-3}\,\mathrm{Pa \cdot s}$), and in absence of H_2O_2. According to Eq. (4) and for low $\kappa \approx 0.008$, changing the viscosity modifies the slope of the critical magnetic field amplitude for the uplift, expressed as $B_0 \propto \beta^2 f^2$. As shown in Fig. 7, effectively we find that all data taken for different ratio η/η_w can be rescaled along a linear curve, black line, as a function of the driving frequency f.

Section S5: Vertical force over the active particle

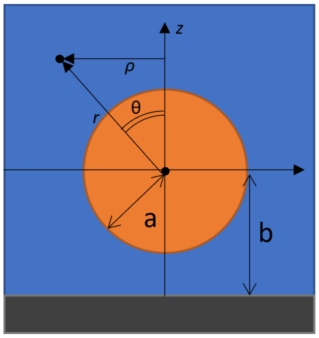

Figure 8: Schematic of an active spherical particle of radius a at an elevation b above a surface.

To illustrate the origin of the vertical diffusiophoretic and diffusioosmotic forces acting on active hematite, we propose a minimal model. We consider a spherical colloid Ω of radius a separated by a distance b from a solid substrate S. We place a permanent point source at the center of the colloid, $\boldsymbol{r} = 0$, that releases a chemical at a rate J and we assume low Péclet number such that advection is negligible compared to diffusion. The concentration field satisfying the corresponding equation, $D\nabla^2 C + J\delta(\boldsymbol{r}) = 0$ reads $C(\boldsymbol{r}) = J/(4\pi D r)$, where D is the diffusivity of the chemical in water and r is the distance from the colloid. To ensure no-flux for the chemical at the substrate, we add a symmetric image source mirrored relative to the surface at $z = -2b$. Using further the cylindrical, (r, θ, z), or spherical, (r, θ, ϕ), coordinates, for the distribution of concentration we finally obtain $C(r, \theta, z) = J/(4\pi D)[(\rho^2 + z^2)^{-1/2} + (\rho^2 + (z + 2b)^2)^{-1/2}]$ or

$$C(r, \theta, \phi) = \frac{J}{4\pi D}\left(\frac{1}{r} + \frac{1}{\sqrt{r^2 + 4br\cos\theta + 4b^2}}\right). \tag{5}$$

The presence of the impermeable substrate breaks the spherical symmetry and generates a concentration gradient along the colloid surface,

$$\boldsymbol{\nabla}_{\parallel} C\big|_{\Omega} = \frac{1}{a}\left.\frac{\partial C}{\partial \theta}\right|_{r=a} \boldsymbol{e}_\theta = \frac{J}{2\pi D}\frac{b\sin\theta}{(a^2 + 4ab\cos\theta + 4b^2)^{3/2}}\boldsymbol{e}_\theta, \tag{6}$$

and we also obtain a radial gradient of concentration along the substrate surface

$$\boldsymbol{\nabla}_{\parallel} C\big|_S = \left.\frac{\partial C}{\partial \rho}\right|_{z=-b} \boldsymbol{e}_\rho = -\frac{J}{2\pi D}\frac{\rho}{\left(\rho^2+b^2\right)^{3/2}}\boldsymbol{e}_\rho, \tag{7}$$

These concentration gradients along the solid surfaces, $\boldsymbol{\nabla}_{\parallel} C$, generate an osmotic slip velocity[7]

$$\boldsymbol{u}_{slip} = \mathcal{M}\boldsymbol{\nabla}_{\parallel} C, \tag{8}$$

where $\mathcal{M}$ the surface mobility. By applying the Lorentz reciprocal theorem,[8] we can express the vertical phoretic force $\boldsymbol{F}_{ph}$ resulting from the slip velocity along the colloidal particle, and the vertical osmotic force $\boldsymbol{F}_{osm}$ resulting from the slip velocity along the substrate. To apply this theorem, it is necessary to first introduce an appropriate dual problem, sharing the same geometry as the osmotic problem but with "appropriate" boundary conditions. In this specific case, it corresponds to a colloid with a velocity $u_\Omega = -\hat{U}\boldsymbol{e}_z$ along its surface. An analytical solution for this dual problem can be found in the literature,[9] originally proposed as a solution for a colloid falling towards a substrate. The expressions for the two forces are then given by

$$\boldsymbol{F}_{ph/osm}\cdot\boldsymbol{e}_z = -\int_{\Omega/S}\frac{\boldsymbol{n}\cdot\hat{\boldsymbol{\sigma}}}{\hat{U}}\cdot\left(\mathcal{M}_{\Omega/S}\boldsymbol{\nabla}_{\parallel} C\right)\,\mathrm{d}S \tag{9}$$

with $\boldsymbol{n}$ being the normal to the surfaces and $\hat{\boldsymbol{\sigma}}$ the viscous stress for the dual problem. The rigorous demonstration of these expressions from the Lorentz reciprocal theorem can be found in a previous work.[10] Knowing that $\hat{\boldsymbol{\sigma}} \propto \hat{\eta}U$ with η being the fluid viscosity, we can verify that the expressions for the forces are independent of $\hat{U}$.

We now can compute numerically the variation of the force ratios $F_{osm}/\mathcal{F}_S$ and $F_{ph}/\mathcal{F}_\Omega$, with $\mathcal{F}_{\Omega/S} = \eta a\mathcal{V}$ and $\mathcal{V} = JM_{\Omega/S}/(Da^2)$ representing the typical osmotic flow velocity. For $M_{\Omega/S}$ negative,[10] both forces contribute to pushing the colloids towards the substrate. In the case of the colloid in contact with the substrate, $b = a$, we find that $F_{osm}/\mathcal{F}_S \approx F_{ph}/\mathcal{F}_\Omega \approx 20$.

Knowing that the phoretic velocity for a spherical colloid is the opposite of the average osmotic

slip velocity on its surface,[11] we deduce that $\mathcal{V}$ is on the order of a few micrometers per second.[12] For $a = 1\,\mu\mathrm{m}$, $\eta = 10^{-3}\,\mathrm{Pa}\cdot\mathrm{s}$, and a density of $5\,\mathrm{g/cm}^3$ for the hematite, we find that the contributions of osmotic and phoretic forces relative to gravity, $(F_{osm} + F_{ph})/F_{grav} \simeq 1 - 10$, as observed in the experiment.

The Lorentz reciprocal theorem is a powerful tool for deriving formulas for forces, but it provides very little information about the underlying mechanisms. For the phoretic force, the impermeable substrate breaks the vertical symmetry, generating a vertical concentration gradient along the particle surface oriented toward the substrate. This induces a surface osmotic flow along the opposite direction for $\mathcal{M}_\Omega < 0$, which in turn pushes the colloid towards the substrate.[11] For the osmotic force, the concentration gradient along the substrate generates a radial and centripetal flow for $\mathcal{M}_S < 0$. Due to incompressibility, this recirculation generates a vertical flow at the center, i.e., on the colloid, pushing it toward the substrate through viscous interactions.

Section S6: Supporting video file

With the article there is one videoclip as support for the main text.

- **Video (.mp4)**: This videoclip is divided into four different videos showing the dynamics of four hematite particles spinning due to an external magnetic field with frequency $f = 10$ Hz and at different field amplitudes such that the particle is in the down (two videos in the top row) or in the up (two videos in the bottom row) states and at different illumination conditions. In the two videos of the first column the light is off ($I = 0$) and the applied magnetic field has amplitude $B_0 = 0.75$ mT (first column, top row; down state), or $B_0 = 1.25$ mT (first column, bottom row; up state). In the two videos of the second column the blue light with wavelength $\lambda = 450 - 490$nm is applied at an intensity $I = 28\,\mathrm{mW\,cm}^{-2}$ and the applied magnetic field has amplitude $B_0 = 1.5$ mT (second column, top row; down state), or $B_0 = 2.0$ mT (second column, bottom row; up state). The videos are all in real time and they correspond to Fig. 1(b) of the main text.

References

(1) Sugimoto, T.; Muramatsu, A. Formation mechanism of monodispersed α-Fe_2O_3 particles in dilute $FeCl_3$ solutions. *J. Colloid. Interf. Sci.* **1996**, 184, 626.

(2) Massana-Cid, H.; Codina, J.; Pagonabarraga, I.; Tierno, P. Active apolar doping determines routes to colloidal clusters and gels. *Proc. Natl. Acad. Sci. USA* **2018**, 115, 10618.

(3) Palacci, J.; Sacanna, S.; Vatchinsky, A.; Chaikin, P. M.; Pine, D. J. Photoactivated colloidal dockers for cargo transportation. *J. Am. Chem. Soc.* **2013**, 135, 15978-15981.

(4) Martinez-Pedrero, F.; Navarro-Argemí, E.; Ortiz-Ambriz, A.; Pagonabarraga, I.; Tierno, P. Emergent hydrodynamic bound states between magnetically powered micropropellers. *Sci. Adv.* **2018**, 4, eaap9379.

(5) Martinez-Pedrero, F.; Massana-Cid, H.; Tierno, P. Assembly and transport of microscopic cargos via reconfigurable photoactivated magnetic microdockers *Small* **2017**, 13, 1603449.

(6) Dhar, P.; Swayne, C. D.; Fischer, T. M.; Kline, T.; Sen, A. Orientations of overdamped magnetic nanorod-gyroscopes. *Nano Lett.* **2007**, 7, 1010-1012.

(7) Marbach, S.; Bocquet, L. Osmosis, from molecular insights to large-scale applications. *Chem. Soc. Rev.* **2019**, 48, 3102.

(8) Masoud, H.; Stone, H. A. The reciprocal theorem in fluid dynamics and transport phenomena. *J. Fluid Mech.*, **2019**, 879, P1.

(9) Ganatos, P.; Weinbaum, S.; Pfeffer, R. A strong interaction theory for the creeping motion of a sphere between plane parallel boundaries. Part 1. Perpendicular motion. *J. Fluid Mech.*, **1980** 99, 739-753.

(10) Boniface, D.; Leyva, S. G.; Pagonabarraga, I.; Tierno, P. Clustering induces switching between phoretic and osmotic propulsion in active colloidal rafts. *Nat. Commun.*, **15**, 5666. (2024).

(11) Stone, H. A.; Samuel, A. D. T. Propulsion of microorganisms by surface distortions *Phys. Rev. Lett.*, **1996**, 77, 4102-4104.

(12) Palacci, J.; Sacanna, S.; Steinberg, A. P.; Pine, D. J.; Chaikin, P. M. Living crystals of light-activated colloidal surfers. *Science*, **2013** 339, 936-940.
